# Synthesis and Characteristics of Double-Shell Mesoporous Hollow Silica Nanomaterials to Improve CO_2_ Adsorption Performance

**DOI:** 10.3390/mi12111424

**Published:** 2021-11-19

**Authors:** Jong-tak Lee, Jae-Young Bae

**Affiliations:** Department of Chemistry, Keimyung University, Daegu 42601, Korea; atomos27@hanmail.net

**Keywords:** double-shell mesoporous hollow silica sphere, CO_2_ adsorption, high pore volume

## Abstract

To improve the adsorption performance of carbon dioxide, which is considered the main culprit of greenhouse gases, the specific surface area and high pore volume of the adsorbing material should be considered. For a porous material, the performance of carbon dioxide adsorption is determined by the amine groups supporting capacity; the larger the pore volume, the greater the capacity to support the amine groups. In this study, a double-shell mesoporous hollow silica nanomaterial with excellent pore volume and therefore increased amine support capacity was synthesized. A core–shell structure capable of having a hollow shape was synthesized using polystyrene as a core material, and a double-shell mesoporous shape was synthesized by sequentially using two types of surfactants. The synthesized material was subjected to a sintering process of 600 degrees, and the N_2_ sorption analysis confirmed a specific surface area of 690 m^2^/g and a pore volume of 1.012 cm^3^/g. Thereafter, the amine compound was impregnated into the silica nanomaterial, and then, a carbon dioxide adsorption experiment was conducted, which confirmed that compared to the mesoporous hollow silica nanomaterial synthesized as a single shell, the adsorption performance was improved by about 1.36 times.

## 1. Introduction

With the industrial revolution, the use of fossil fuels has rapidly increased, resulting in a gradual increase in carbon dioxide generation, which has had a significant impact on the atmospheric environment [1,2,3]. Consequently, the impacts of global warming are becoming more serious, and carbon dioxide adsorption has become an important research subject worldwide [4,5,6,7,8].

Currently, there is active research on adsorption using porous materials, such as zeolite [9,10,11], activated carbon [12,13,14,15], metal–organic frameworks (MOFs) [16,17,18,19], and mesoporous silica with amine functional groups [20,21,22,23]. Among them, mesoporous silica has shown the high specific surface area and tremendous porous characteristics compared to other materials, and it is able to introduce various functional groups due to the presence of numerous hydroxyl (-OH) groups on the surface. Owing to these characteristics, mesoporous silica has demonstrated excellent gas adsorption properties [24]. In addition, the mesoporous silica with an amine functional group shows excellent carbon dioxide adsorption properties, even in a moist environment; the amine group on the silica surface combines with carbon dioxide even in the presence of moisture to form a hydrogen carbonate salt, so that the porous silica can adsorb CO_2_ [25]. That is, in the case of porous silica with an amine functional group, it has the advantage of supplementing the water stability, which was lacking in the other existing materials, and it was identified as a disadvantage [26].

So far, various studies on how to improve the physical and chemical properties of mesoporous silica have been conducted. Especially, the research on mesoporous hollow silica, which is known to have a unique structure, has attracted a lot of interest [27]. Commendably, the advantage of providing a specific surface area and a three-dimensional pore structure by synthesizing a porous hollow silica shell enables improvement in the carbon dioxide adsorption performance. Therefore, the aim of the present study was to further increase the advantages of the mesoporous hollow silica through a double-shell mesoporous synthesizing method. In addition, the carbon dioxide adsorption performance of a double-shell mesoporous hollow silica is compared with that of the single shell.

## 2. Experimental Section

### 2.1. Materials

Styrene (≥99%, Sigma–Aldrich, Burlington, MA, USA) was used as a core to make a hollow structure, and 2,2’-azobis (2-methylpropionamidine) dihydrochloride (AIBA, 97%, Sigma–Aldrich, Burlington, MA, USA) was used as an initiator for the polymerization of styrene. To provide the porosity of the hollow silica, cetyltrimethylammonium chloride (CTACl, 25 wt %, Sigma–Aldrich, Burlington, MA, USA) and surfactant of poly(ethylene glycol)-block-poly(propylene glycol)-block-poly(ethylene glycol) (P123, Sigma–Aldrich, Burlington, MA, USA) were used, tetraethylorthosilicate (TEOS, 98%, Sigma–Aldrich, Burlington, MA, USA) was used as a silica precursor, and aqueous ammonia (NH_4_OH, (25–30)%, Duksan, Seoul, Korea) and hydrochloric acid (HCl, (35–37)%, SAMCHUN, Seoul, Korea) were used for pH adjustment. Tetraethylenepentamine (TEPA, 93%, Kanto Chemical, Tokyo, Japan) was used to introduce an amine group into the synthesized mesoporous hollow silica.

### 2.2. Polystyrene Synthesis

Polystyrene is required as a template to synthesize the mesoporous hollow silica. For the synthesis of polystyrene as a template, first, 20 mL of styrene was added to 600 mL of water in a three-necked flask, and then, it was stirred at 400 rpm. After stirring at room temperature (RT) for 30 min, 2.0 g of AIBA was added, and the temperature was raised to 95 degrees and stirred for 18 h; then, the solution turned white, and spherical polystyrene was synthesized.

### 2.3. Synthesis of Mesoporous Hollow Silica

First, 3400 mL of a solvent obtained by mixing distilled water and ethanol in a 4:3 volume ratio was added into 600 mL of the synthesized polystyrene solution and stirred. After stirring at RT for 30 min, 30 mL of CTACI was added, which was followed by stirring at RT for 10 min, and 60 mL of TEOS, a silica precursor, was added. After adding the TEOS, the mixture was stirred at RT for 30 min, and then aqueous ammonia was added dropwise until the pH was 9, which was followed by stirring at RT for 15 h. After the stirring was completed, the solution was precipitated and settled. Then, the material was filtered, washed three times with distilled water, and dried in an oven at 80 °C for 24 h. After the dried sample was finely ground and calcined at 600 °C for 5 h, mesoporous hollow silica material (MHS) was synthesized.

For comparison, mesoporous hollow silica (PHMS) using P123 was synthesized. After adding the same amount to the synthesized polystyrene solution at the same solvent ratio, hydrochloric acid was added dropwise until the pH was pH = (3–4). After the addition of hydrochloric acid, 50 mL of P123 was added, stirred at RT for 3 h, and then 60 mL of TEOS, a silica precursor, was added, and stirred at RT for 30 min. Next, ammonia water was added dropwise until the pH was 9, and it was stirred at RT for 15 h. After the stirring, the solution was precipitated and settled. The material was filtered, washed three times with distilled water, and dried in an oven at 80 °C for 24 h.

### 2.4. Synthesis of the Double-Shell Mesoporous Hollow Silica

**Scheme 1:**
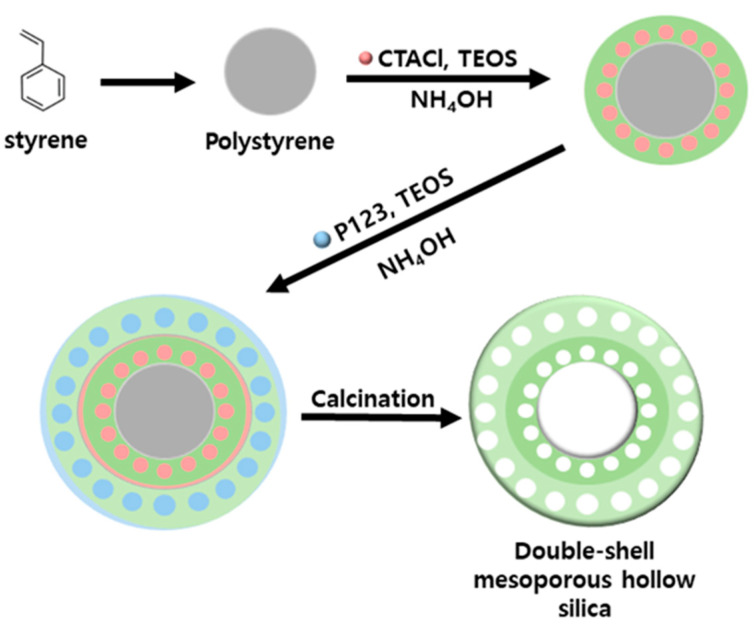
Synthesis scheme of double-shell mesoporous hollow silica.

To synthesize double-shell mesoporous hollow silica, 3400 mL of the solvent obtained by mixing distilled water and ethanol in a 4:3 volume ratio was added into 600 mL of the synthesized polystyrene solution and stirred. After stirring at RT for 30 min, 30 mL of CTACI was added, which was followed by stirring at RT for 10 min, and 40 mL of TEOS, a silica precursor, was added. After adding the TEOS, the mixture was stirred at RT for 30 min, and then aqueous ammonia was added dropwise until the pH was 9, which was followed by stirring at RT for 15 h. After the stirring, 20 mL of P123, a nonionic surfactant, was continuously added without filtering, and the stirred solution was dried. Next, HCl was added until the pH was pH = (3–4), to form a pH range for constituting the micelles of P123. Then, 20 mL of TEOS, a silica precursor, was added, the solution was stirred at RT for 30 min, and then aqueous ammonia was added dropwise until the pH was 9. The resulting solution was stirred at RT for 15 h to obtain a precipitate, which was washed 3 times with distilled water and then calcined at 600 °C for 5 h. Then, the double-shell mesoporous hollow silica material (DMHS) was synthesized. This synthesis process is shown in Scheme 1.

### 2.5. Introduction of the Amine Group

The synthesized mesoporous hollow silica (MHS, PMHS) and double-shell mesoporous hollow silica (DMHS) were used as amine supporters for CO_2_ adsorption. Each 0.5 g of the synthesized MHS, PHMS, and DMHS were added into 50 mL of absolute ethanol, respectively, and dispersed by stirring at RT for 30 min. After adding 1 g of TEPA into each dispersed solution and stirring at RT for 1 h, each solvent was removed from the solution by stirring at 50 °C for 20 min using a rotary evaporator. After the removal of the solvent, each sample was dried in an oven at 100 °C for 1 h to obtain MHS, PHMS, and DMHS introduced with amine groups.

### 2.6. Characterization

Transmission electron microscopy (TEM; JEM-2100F) was used to analyze the shape, structure, size, and shell thickness of the polystyrene, the mesoporous hollow silica, and the double-shell mesoporous hollow silica materials. After dispersing the sample in an ethanol solution, a small amount of the colloidal solution was collected on a copper grid, dried, and used as a sample. When measuring the sample, the accelerating voltage was 200 kV, and the analysis was conducted with the resolution of 0.25 nm at maximum N_2_ sorption. QUANTACHROME (Qudrasorb SI, Anton Paar, Graz, Austria) was used to determine the specific surface area and the pore distributions of the mono-shell mesoporous hollow silica and double-shell mesoporous hollow silica. The measurements were carried out conducted at 77 K using liquid nitrogen, and the adsorbed nitrogen was normalized to standard temperature and pressure. Prior to analysis, moisture and impurities adsorbed on the surface were removed through heat treatment at 200 °C for 3 h. The Brunauer–Emmett–Teller (BET) specific surface area was calculated from the linear part (P/P_0_ = (0.05–0.30) of the BET equation, and the volume and size of the pores were calculated using the Barrett–Joyner–Halenda (BJH) equation. Fourier transform infrared spectroscopy (FTIR; thermo, Nicolet iS50) was used to confirm whether functional groups were attached to the double-shell mesoporous hollow silica introduced with the amine groups. Measurements were carried under atmospheric environment, and the sample was in a powder state. Energy-Dispersive X-ray Spectroscopy (EDS; JEOL, Tokyo, Japan, EX-746OOU4L2Q) was used for elemental distribution analysis of the double-shell mesoporous hollow silica introduced with the amine groups. At analysis, the Working Distance (WD) was adjusted to 9.5–10.5 mm, and to ensure reliability, the Counts Per Second (CPS) was set to 300,000 or more during the total analysis time. To confirm the gas adsorption capacity of the mono-shell and double-shell mesoporous hollow silica introduced with the amine groups, the carbon dioxide adsorption gas chromatography (GC; HP 6890) equipped with a thermal conductivity detector (TCD) was prepared. The adsorbed gas was mixed with nitrogen as a base and a carbon oxide concentration of 30%. The gas flow rate was 5 mL/min through a mass flow controller (MFC), and high-purity helium gas was used as the make-up gas.

## 3. Results and Discussion

**Figure 1:**
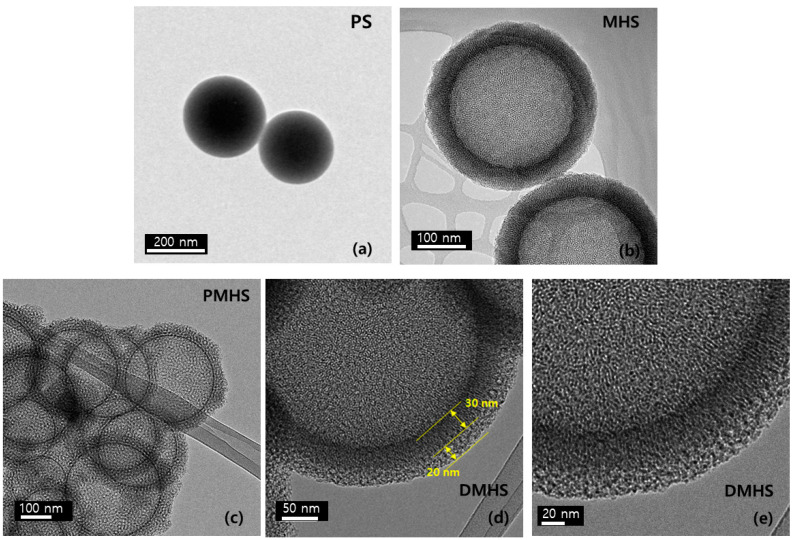
TEM images of the (**a**) PS, (**b**) MHS, (**c**) PHMS, and (**d**,**e**) DMHS.

Figure 1 shows TEM images of the polystyrene, mesoporous hollow silica, and double-shell mesoporous hollow silica, which were synthesized in a spherical shape. Figure 1a confirms that polystyrene is approximately 270 nm in shape and has a perfect spherical shape. Figure 1b–d show TEM images of MHS, PMHS, and DMHS after polystyrene and surfactant removal, and Figure 1e shows an enlarged TEM image of Figure 1d. The hollow size in Figure 1b–d was 270 nm, which is the same size as that of polystyrene, and the shell thicknesses of MHS, PMHS, and DMHS were 45, 45, and 50 nm, respectively. In particular, in DMHS, the thickness of the primary shell was 30 nm, and the thickness of the secondary shell was 20 nm (Figure 1d). As the PS used was the same size, all three samples had the same hollow size. The pore size of the shell in MHS was small due to the use of CTACl, but the pore size of the PMHS shell using P123 was relatively large. Furthermore, it was observed that the primary shell of DMHS forms small pores, and the secondary shell forms large pores; this is because the molecular weight of P123 is larger than that of CTACI, which made relatively large micelles, and it was removed by calcination [28]. 

**Figure 2:**
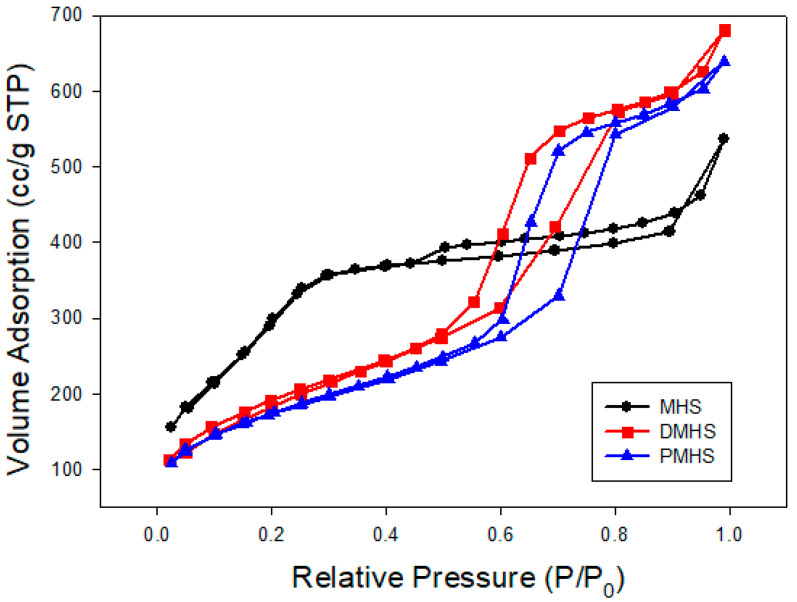
N_2_ adsorption–desorption isotherms for MHS, PMHS, and DMHS.

Figure 2 shows the N_2_ adsorption–desorption isotherm graphs of MHS, PMHS, and DMHS. All three samples of the MHS, PMHS, and DMHS showed type IV adsorption isotherm with an H2 hysteresis loop.

The adsorption and desorption curves were separated at the point where the relative pressure was 0.5, therefore confirming the mesoporous morphology. The BET-specific surface area of MHS was 1164 m^2^/g, and those of PHMS and DMHS were 609 and 690 m^2^/g, respectively. Although there was a significant difference in the specific surface area, PHMS and DHMS had higher volumetric adsorption values in the N_2_ adsorption–desorption isotherm graphs, indicating superior pore formation than MHS.

**Figure 3:**
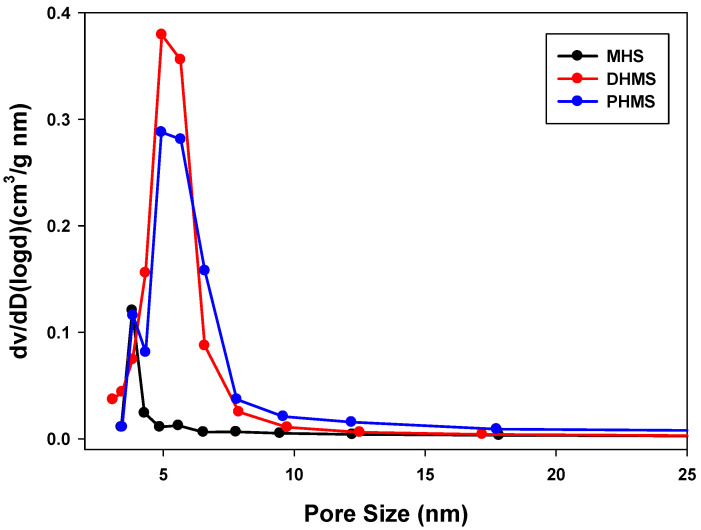
Pore size distribution of MHS, PMHS, and DMHS.

Figure 3 shows the pore size distribution of MHS, PHMS, and DHMS. As predicted from the N_2_ adsorption–desorption isotherm graphs, PHMS and DHMS have wider pore sizes and larger pore volumes than MHS, while DHMS has a larger pore volume than PHMS; this is because double shells with different pore sizes can form larger pores than single shells [29]. This may mean that as the internal pore volume is large, there is sufficient capacity to support more amine groups [30]. Table 1 summarizes the BET-specific surface area, pore volume, and pore sizes of MHS, PHMS, and DHMS.

**Figure 4:**
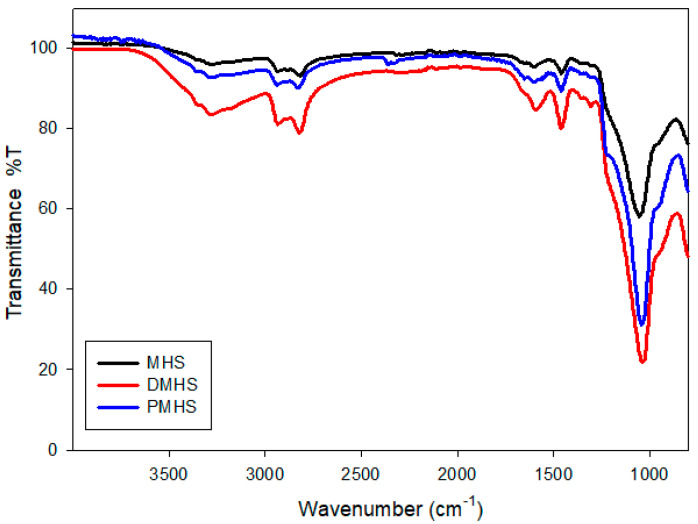
Infrared spectra of the MHS, PHMS, and DMHS after the amine modification.

Figure 4 shows the FTIR measurements after adding amine groups to the three types of mesoporous hollow silica, MHS, PMHS, and DMHS. The symmetry of N–H bonding and the asymmetric stretching oscillations are observed at 3500–3300 cm^−1^, and the symmetry of C–H bonding and the asymmetric stretching oscillations were observed at 3000–2800 cm^−1^. In addition, C–H shear oscillation and N–H bending oscillation in amine were observed at 1457 and 1596 cm^−1^ [31]. It was confirmed that the intensity of this peak increased from MHS to DMHS. From this, it is confirmed that the number of amines supported on the mesoporous silica were in the order of MHS < PHMS < DMHS. The elemental analysis result of EDS confirmed the actual supported amount of amine groups, as shown in Table 2. Similar to the N–H peak intensity trend of the FTIR graph, the nitrogen content was also the highest in DMHS. Therefore, DMHS, which has the largest pore volume, supports more amines than the other samples [30]. For more accurate confirmation, Table 3 compares the amounts of accommodated amines by comparing the weight of the sample before and after adding amines. The amine-supporting efficiency was calculated as shown in Equation (1). Table 3 confirmed that the number of amines increased with increasing pore volume. This was consistent with the FTIR and EDS analysis.

(1) $$\frac{\mathrm{After}\;\mathrm{amine}\;\mathrm{modification}\;(g)\;-\;\mathrm{Before}\;\mathrm{amine}\;\mathrm{modification}\;(g)\;}{\mathrm{Before}\;\mathrm{amine}\;\mathrm{modification}\;(g)}\times 100\;$$

**Figure 5:**
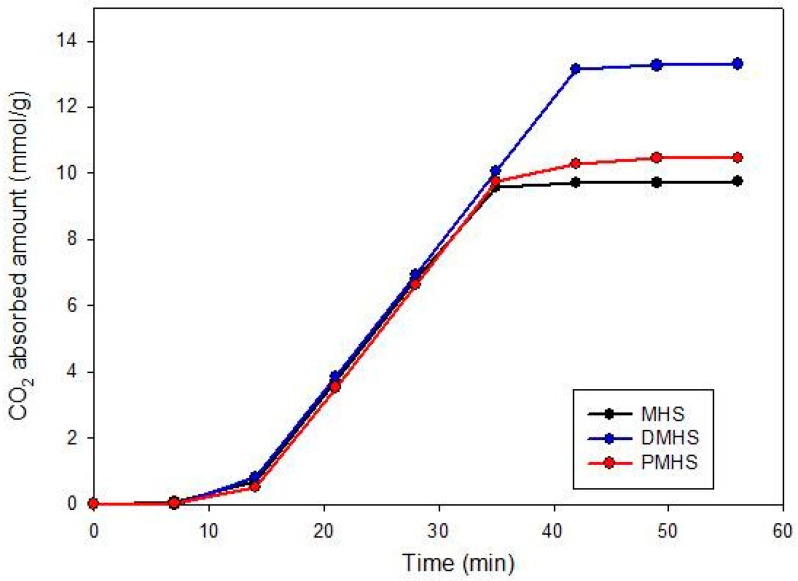
CO_2_ adsorption performance of MHS, PHMS, and DMHS.

Figure 5 shows the CO_2_ adsorption performance of the MHS, PMHS, and DMHS, which are amine-supported mesoporous hollow silica. The amine group is one of the functional groups that has CO_2_ adsorption performance; to have excellent CO_2_ adsorption performance, a large amount of amine should be supported on the mesoporous hollow silica. Figure 5 shows that DMHS supported the largest amount of amines and therefore the highest CO_2_ adsorption efficiency of 13.296 mmol/g, and PHMS supported the second largest amount of amine and had an adsorption efficiency of 10.450 mmol/g. Based on these findings, to improve CO_2_ adsorption, the amount of the amine supported on the mesoporous silica should be increased; for that, the pore volume of the mesoporous silica is needed for the increased amount of the supported amine. When the pore volume is large, the amine is often located both inside and outside the support. However, Yan et al. reported that the adsorption efficiency of amines located inside the support was better than that of those located outside [32,33]. Table 4 summarizes the results.

## 4. Conclusions

In this study, double-shell mesoporous hollow silica (DMHS) with excellent pore volume was synthesized. The synthesized DMHS was introduced into an amine functional group via a support method to have a selectivity for CO_2_ adsorption. The synthesized DMHS can be used as a solid amine support for carbon dioxide adsorption; since it has a larger pore volume than single-shell mesoporous hollow silica, a large amount of amine can be introduced. This is because the high pore volume of DMHS allowed the introduction of amine groups inside and outside of the mesoporous hollow silica. This ensured a high carbon dioxide adsorption efficiency of 13.296 mmol/g, which was 1.36 times higher than that of MHS synthesized with CTACI, and it showed a higher amount of CO_2_ adsorption than other CO_2_ adsorption materials. This double-shell mesoporous silica has a high pore volume and therefore can support an increased amount of functional groups. This makes it ideal for CO_2_ adsorption studies and also for various adsorbents and catalyst studies.

## Figures and Tables

**Table 1:** The textural properties of the MHS, PHMS, and DMHS.

Sample Name	Surface Area (m^2^/g)	Pore Volume (cm^3^/g)	Average Pore Size ^a^ (nm)
MHS	1164	0.321	3.309
PHMS	609	0.933	5.679
DMHS	690	1.012	4.944

Note: ^a^ Diameter of the window, determined from the desorption branch according to the BJH method.

**Table 2:** EDS spectra of MHS, PHMS, and DMHS.

Sample Name	Pore Volume (cm^3^/g)	Atom Si (%)	Atom N (%)	Atom C (%)	Atom O (%)
MHS	0.321	12.35	7.97	44.17	35.51
PMHS	0.933	18.65	8.33	37.21	35.81
DMHS	1.012	10.82	13.30	44.27	31.60

**Table 3:** Amine modification percentage of MHS, PHMS, and DMHS.

Sample Name	Before Amine Modification (g)	After Amine Modification (g)	Amine Modification Percent (%)
MHS	0.5	0.8897	77.94
PHMS	0.5	0.9097	81.94
DMHS	0.5	0.9984	99.68

**Table 4:** CO_2_ adsorption amount according to pore volume and amine modification percentage.

Sample Name	Pore Volume (cm^3^/g)	Amine Modification Percent(%)	CO_2_ Capacity (mmol/g)
MHS	0.321	77.94	9.757
PHMS	0.933	81.94	10.450
DMHS	1.012	99.68	13.296

## Data Availability

No new data were created or analyzed in this study. Data sharing is not applicable to this article.
